# Recent advances in mRNA-LNP therapeutics: immunological and pharmacological aspects

**DOI:** 10.1186/s12951-022-01478-7

**Published:** 2022-06-14

**Authors:** Seyed Hossein Kiaie, Naime Majidi Zolbanin, Armin Ahmadi, Rafieh Bagherifar, Hadi Valizadeh, Fatah Kashanchi, Reza Jafari

**Affiliations:** 1 Department of Formulation Development, ReNAP Therapeutics, Tehran, Iran; 2 Student Research Committee, Tabriz University of Medical Sciences, Tabriz, Iran; 3 Nano Drug Delivery Research Center, Kermanshah University of Medical Sciences, Kermanshah, Iran; 4 Experimental and Applied Pharmaceutical Sciences Research Center, Urmia University of Medical Sciences, Urmia, Iran; 5 Department of Pharmacology and Toxicology School of Pharmacy , Urmia University of Medical Sciences , Urmia, Iran; 6 Department of Chemical & Materials Engineering, The University of Alabama in Huntsville, Huntsville, AL 35899 USA; 7 Drug Applied Research Center, Faculty of Pharmacy, Tabriz University of Medical Sciences, Tabriz, Iran; 8 School of Systems Biology, Laboratory of Molecular Virology, George Mason University, Discovery Hall Room 182, 10900 University Blvd, Manassas, VA 20110 USA; 9 Cellular and Molecular Research Center, Cellular and Molecular Medicine Institute, Urmia University of Medical Sciences, Urmia, Iran

**Keywords:** Lipid nanoparticles, mRNA delivery, Immunogenicity, Pharmacologic response, Dendritic cell, Immune system, Toll-like receptor

## Abstract

In the last decade, the development of messenger RNA (mRNA) therapeutics by lipid nanoparticles (LNP) leads to facilitate clinical trial recruitment, which improves the efficacy of treatment modality to a large extent. Although mRNA-LNP vaccine platforms for the COVID-19 pandemic demonstrated high efficiency, safety and adverse effects challenges due to the uncontrolled immune responses and inappropriate pharmacological interventions could limit this tremendous efficacy. The current study reveals the interplay of immune responses with LNP compositions and characterization and clarifies the interaction of mRNA-LNP therapeutics with dendritic, macrophages, neutrophile cells, and complement. Then, pharmacological profiles for mRNA-LNP delivery, including pharmacokinetics and cellular trafficking, were discussed in detail in cancer types and infectious diseases. This review study opens a new and vital landscape to improve multidisciplinary therapeutics on mRNA-LNP through modulation of immunopharmacological responses in clinical trials.

**Graphical Abstract:**

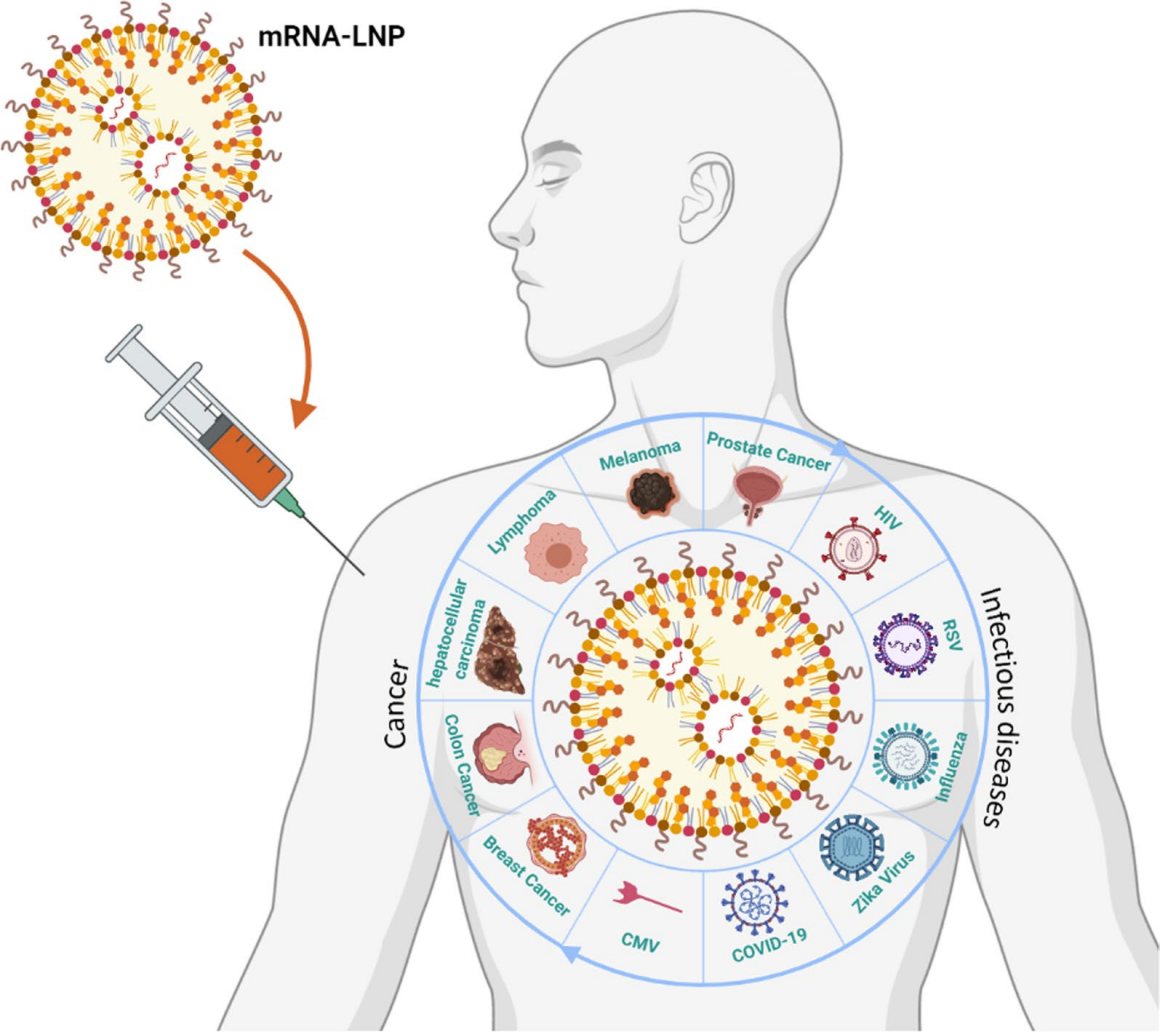

## Introduction

Therapeutics based on RNA holds great promise to expand new drug candidates into developing of translational medicine [1, 2]. This proof of concept study has indicated the broad potential application of cancer and disease treatment and encompassed more than a dozen RNA therapies being tested in clinical trials [3, 4]. ONPATTRO™ (patisiran) is the first of short interfering RNA (siRNA)-based therapeutics and the only FDA-approved RNA interference (RNAi) drug, which has been administrated for the treatment of the transthyretin induced amyloidosis in adult patients [5]. siRNA interferes with specific genes expression through degrading messenger RNA (mRNA) after transcription to prevent the translation [6]. In contrast, mRNA has been utilized in the systemic administration of genetic vaccines in cancer [7] and infectious disease [8] and loading and activation of dendritic cells (DC) for the cellular vaccines [9, 10]. In other words, applications of mRNA have encompassed protein replacement therapy, cancer immunotherapy, vaccines, cellular reprogramming, and gene editing. Usage of aberrant protein for muscular dystrophy demonstrated necessitates mRNA for protein replacement [11] due to mRNA as a single molecule creates many copies of a protein during a short period. Compared to traditional vaccination, mRNA-based vaccines were efficient alternatives for personalized and prophylactic trials such as influenza, human immunodeficiency viruses (HIV), Zika, Cytomegalovirus3 (CMV) 3, and rabies [8, 11–13]. In cancer immunotherapy, in addition to mRNA encodes tumor-associated peptide antigens (Ags) to induce prophylactic immunity like vaccines [14, 15] mRNA modifies T cells with chimeric antigen receptor (CAR) T cell [16, 17]. Gene editing uses mRNA encoding to deliver clustered regularly interspaced short palindromic repeats (CRISPR) transcription activator-like effector nuclease (TALEN) and zinc-finger nucleases (ZFNs) through genome cut-and-paste mechanism for the lasting cure for genetic diseases [18]. The last application of mRNA is cellular reprogramming, which involves programming cell fate and function through tissue engineering and regenerative medicine, for instance, usage four using factors to induce pluripotent stem cells (iPSCs) stem lead to Nobel Prize-winning discovery in 2012 [19].

mRNA-based cancer therapy requires the transfection of antigen-presenting cells (APCs). To develop these vaccines, the transfection of APCs, such as DCs, is required to generate a cytotoxic T-cell response [9]. mRNA does not need any required nuclear localization or transcription but also includes at least a possible section of genomic integration for transfected sequences [20]. Although the mRNA therapeutics have been clinically advanced due to the design and manufacture of mRNA, the broad application of mRNA to express the desired protein is due to in vivo delivery challenges such as nucleases degradation, lack of stability, endosomal trapping, and immunotoxicity responses of mRNA are hindered. Among them, the critical factors of mRNA therapeutic becoming a viable clinical option based on dispelling mRNA instability and nucleases degradation and reducing endosomal trapping result in ineffective delivery to specific target cells through specific-purpose vectors [21–23]. In the last decade**,** the application and development of non-viral or synthetic and viral or natural delivery systems as the most promising strategies for gene manipulation to produce a human protein with higher expression efficiency and lower immunogenicity has attracted enormous interest [24, 25]. Immunogenicity and cytotoxicity concerns, the risks of reverse genome insertion, transient gene expression control, and vector-size limitations of viral constructs restricted their utilization for long-term therapeutics despite the obvious advantage of efficient delivery due to high-efficiency transfection of host cells and low and off-target expression [26, 27].

In contrast, non-viral vectors reduce the main drawbacks of using a viral vector, including innate immune stimulation and cytotoxicity concerns. In addition, non-viral vectors prevent nonspecific interactions with proteins or non-target cells and promote efficient transport to multiple tissues through targeted delivery [28–30]. More recently, numerous non-viral vectors, including polymer [31–33], hybrid [34–36], and even scaffold-mediated [37] nanoparticles (NPs), have been used and fostered for mRNA delivery. Recent studies have demonstrated that lipid-based NPs as the most frequently used carrier for mRNA delivery, are promising to treat cancer and disease disorders [11, 14, 38–40]. The first-time liposome was used for the mRNA delivery system in 1978 [41], and following that, the efforts were gradually continued up to now [42–44]. Finally, lipid nanoparticles (LNP) were developed for mRNA delivery in 2015 to decrease toxicity and immunogenicity concerns and also achieve high in vivo transfection [42].

LNP have mainly consisted of four lipid components such as phospholipids, including especially 2-Distearoyl-sn-glycero-3-phosphocholine (DSPC) or 1,2-Dioleoyl-sn-Glycero-3 Phosphatidylethanolamine (DOPE) and helper lipids such as ionizable amine-containing lipidoid, cholesterol, and polyethylene glycol (PEG) [45–48]. LNP holds tremendous potential for mRNA delivery due to protection from nucleases, avoiding the mononuclear phagocyte system (MPS), low immunogenicity and endosomal trapping, and facilitating cellular uptake [26, 49]. However, LNP loaded with mRNA faced many challenges, including immune interaction (innate and adaptive immunity) such as interplay with LNP components [50]. On the other hand, the LNP compositions indicated a critical challenge for the cytosolic delivery of mRNA to target tissues in the clinic. In some cases, they could enhance DC uptake of the vaccine and prevent the mRNA from interacting with non-APCs, consequently hinder undesirable side effects [51, 52].

Following the onset of the ongoing global coronavirus disease 2019 (COVID-19) pandemic and vaccination in 2020, two mRNA-LNP vaccine platforms from Pfizer/BioNTech, and Moderna were approved and administered worldwide. The lack of explanatory data analysis of the immune responses and pharmacological interplay between LNP used in mRNA-based therapeutics and the target site [53–55], the necessity to evaluate an immunopharmacological profile of LNP have pointed out. This review study discusses the interplay between immune responses and LNP compositions and the profile of immune and pharmacologic responses to delivery mRNA through LNP. In order to analyze the immune responses, the interaction of LNP with neutrophil (Neut), macrophage (MQ), DC, complement, and adaptive immune system will be evaluated, then immunogenicity of mRNA delivery will be studied. Furthermore, pharmacological aspects of mRNA delivery using LNP containing pharmacokinetics (PK) and pharmacodynamics (PD) studies and the role of pattern recognition receptor (PRR) as the most important player in the common cancer cases including prostate, melanoma, lymphoma, hepatocellular carcinoma, colon cancer, and breast cancer will be discussed in detail.

## Interplay of immune responses with compositions of LNP

LNP compositions consisted of cationic or ionizable lipids and helper lipids such as DSPC, DOPE, 1-palmitoyl-2-oleoyl-sn-glycero-3-phosphoethanolamine (POPE), cholesterol, and PEG lipids. The cationic lipid in liposomal formulation for spontaneous negatively charged mRNA encapsulation was utilized by a combination of attractive electrostatic and hydrophobic interactions [56, 57]. Cholesterol as neutral lipid not only enhances particle stability and membrane fusion [58] but also promotes transfection efficiency in vitro [59] and in vivo [60, 61]. Although, it has been demonstrated that lipoplexes dissociate rapidly by substituting cholesterol with DOPE due to serum binding [58, 59]. Moreover, cholesterol can improve CL-complexed mRNA [62] and enhance transfection efficiency of mRNA delivery on account of the physical stability of the colloidal dispersion [63–66] in contrast, excess cholesterol destructs liposome preparation [62]. Using cationic lipids for in vivo administration demonstrated relative toxic effects due to high positive charge and poor endosomal escape [67]. Furthermore, they act to trigger an immunological response through the antibodies production. Forasmuch as helper lipids can shield the positive charge of lipoplexes, decrease accumulation in the lung, shifting in other organs specific the liver [68]. Furthermore, helper lipids can induce systemic inflammation due to their uptake by the liver kupffer cells [30, 42, 69]. DSPC, which is substrates for phospholipases A2 (PLA2s), plays a vital role in releasing eicosanoids (proinflammatory mediator) and produces allergens that induce inflammatory cytokines. Moreover, it is reported that the lysosomal PLA2s and cytosolic PLA2s may cause complement activation and anaphylaxis, respectively [70, 71]. To bypass these problems, reduce positive charge, overcome endosomal escape, and decrease immune and inflammatory responses, ionizable lipids for mRNA delivery were developed [72–74].

Despite common lipid materials that reactive T cells through the restriction CD1 family of APCs by the T cells [75, 76], phospholipids (PLs) generally do not incorporate reactive T cells [77]. Following in vivo administration of the first dose of phosphatidylcholine **(**PC) lipids, B-1 cells directly activate. For this reason, they induce natural Immunoglobulin M (IgM). Following repeated dosing, delivery to APC, including follicular DCs and marginal zone (MZ) B cells, was pushed due to anti-PC IgMs binding to LNP [78]. Cruz-Leal et al. showed the rational evidence for interaction between B-1 cells and dipalmitoyl-phosphatidylcholine (DPPC)-liposomes with ovalbumin (OVA) entrapped. This liposomal formulation modulates the immune response to OVA, which plays an antigen role in evaluating the character of B-1 cells in humoral immunity [78]. Furthermore, they demonstrated that following immunization of BALB/c mice with DPPC-liposomes containing OVA, B-1 cells secrete DPPC-specific IgM independently of the presence of OVA [79].

In 1989, one year after that, Felgner et al. introduced LNP, including 1,2-di-O-octadecenyl-3-trimethylammonium (DOTMA) and DOPE for nucleic acid delivery transfection; LNP was assessed with luciferase mRNA for in vivo analysis (human, mouse, rat, drosophila, and Xenopus) [80, 81]. DOPE, which is synthetic, unsaturated phosphatidylethanolamine (PE) as a common class of membrane or neutral lipid, increases the efficiency of LNP considerably. In contrast to other neutral PLs of the same acyl chain composition, such as even dioleoylphosphatidylcholine (DOPC), with the most similar in structure*,* DOPE serves to promote intracellular delivery of RNA [82–84] due to an endosmotic or fusogenic role, similar to the adenovirus [85] and fusion peptides [86]. This unique performance is relevant to the known relation between unsaturated PE, non-bilayer lipid structures, and membrane fusion [87]. Based on rational connectedness conjecture, DOPE is responsible for enabling LNP/RNA cargo to fuse with either the plasma or endosomal membrane after phagocytosis [88]. First-time, Zuhorn et al. indicated the major entry pathway to transfect lipoplexes (SAINT-2/DOPE) was clathrin-mediated endocytosis (CME) [89]. DOPE disrupts lipid bilayers to non-bilayer hexagonal (HII) at acidic pH because of its geometry with two bulky and unsaturated oleoyl chains, which make a cone-like shape [90, 91]. Moreover, the enriched level of DOPE could destabilize the endosome membrane; thus, the LNP could release mRNA into the cytosol after the fusion [90]. To that end, excessive fusogenicity leads to an enhanced interaction with serum proteins [88].

In this study, mRNA encoding the HIV-1 antigen gag with LNP (1,2-dioleoyl-3-trimethylammonium-propane (DOTAP)/DOPE) could be delivered to DCs for immunization shows antigen-specific responses which display immune-activating properties such as secretion of type I interferon (IFN). IFN inhibits DOTAP/DOPE loaded antigen-encoding mRNA expression and results in the induction of antigen-specific immune responses [43]. On the other hand, although some studies showed DC maturation and Toll-like receptor (TLR) agonists restrict mRNA transfection, therefore decreased overall expression could be happened [92, 93]. To evaluate the effect on co-delivery of modified mRNA with nucleoside and TLR agonists to induce effective antigen-specific T cell immunity for improving mRNA vaccination efficiency was studied, and cholesterol was replaced by DOPE in lipoplex formulation. In what follows, the stability and transfection of APCs by DOTAP-cholesterol mRNA lipoplexes was successful; however, the mRNA expression was hindered by induction of cellular entry type I IFNs through unmodified mRNA. In contrast, modified mRNA with nucleoside declines the intracellular immune recognition and release of type I IFN [94].

In order to remove intolerable side effects of cationic lipids such as pro-inflammatory responses, toxicity, and immunogenicity following systemic administration, PEGylated PLs such as methoxy-PEG-distearoyl phosphoethanolamine (mPEG-DSPE) and dimyristoyl-rac-glycero-3- methoxy-PEG (DMG-mPEG) have been added to the membrane of the cationic lipids on the LNP surface. Moreover, PEGylation of LNP due to high water uptake creates a steric barrier around the LNP, which prevents the aggregation of LNP and inhibits LNP from reticuloendothelial system (RES) recognition. Despite these advantages, PEG plays a double-edged sword role. It inhibits the conversion of the bilayer to hexagonal form and eliminates targeted cellular uptake due to biodistribution reduction. Thus, PEGylation prolongs the circulation half-life, improves transfection efficiency and bioavailability, and decreases MQ uptake of LNP [95–98]. Following the first administration, the secretion of anti-PEG-IgM, which is responsible for the accelerated blood clearance (ABC), results in the more rapid clearance of LNP [99, 100]. Similarly to Cruz-Leal group researches which PC lipids had directly activated B-1 cells to induce natural IgM [78, 79], recently it demonstrated that interaction between B-1 cells and LNP leads to anti-PEG IgM response, both humoral responses being critical in driving the clearance of the LNP from the blood and into the MPS of the lymphoid (and other) tissues. Extending these findings to other delivery technologies that might similarly be subject to natural IgM opsonization is tempting. For instance, gene therapies often rely on viral vectors that include repeating PC-like epitopes and other oligonucleotide therapeutics that use LNP like those described in this study [15, 40].

## Impact of LNP characterization and immune responses

Some physical and chemical properties of LNP such as size, polydispersity index (PDI), surface chemistry, charge and molar ratio of components affect their interaction with the immune system and biodistribution [2, 101]. Few studies have shown the impact of LNP characterization and immune responses up to now. In 2021, Hassett et al. investigated the effect of various biophysical factors on the immunogenicity of the CMV mRNA-LNP vaccine through the determination of antibody titer. Among the different parameters, LNP size showed the highest correlation with immunogenicity. It was observed that in mice, there is a positive relationship between size and immunogenicity; as the LNP size increases (until 100 nm), the antibody titer increases. While in non-human primates (NHPs), immunogenicity is independent of the size of the LNP, and the robust immune response is generated in all particle sizes studied [102]. Brewer et al. evaluated the antibody response to OVA-loaded liposomes with different sizes (100, 155, 225, and 560 nm) in mice. Significantly higher IgG2a and more significant IFN-γ generation were obtained for larger vesicles, while all other vesicles elicited anti-OVA IgG1 [103]. Similar observations were obtained in another study in which liposomes of either 250 or 980 nm in size were loaded with influenza A hemagglutinin [104]. Liposome size also affects the T-helper 1/T-helper 2 (Th1/Th2) ratio. So that vesicles with a size of 250–750 nm induce more potent Th1 responses compared to larger or smaller vesicles. Multilamellar vesicles, on the other hand, may induce more Th2 responses [103, 104].

In addition, the surface charge of LNP has been shown to affect their ability to stimulate the immune system. For instance, vesicles with a positive or negative charge induce antibody-neutralizing responses greater than neutral vesicles [105]. Cationic lipids increase the immunogenicity of LNP-formulated mRNA vaccines due to their ability to interact with the components of the innate immune system, which in turn leads to acceptable therapeutic efficacy [106]. For instance, cationic lipid dimethyl-dioctadecyl ammonium (DDA) acts as an adjuvant in mRNA-based vaccines due to its ability to induce ionic interactions with Ags [107–109]. Korsholm et al. demonstrated that the absorption of Ags onto DDA liposomes leads to enhanced immunogenicity due to an increase in its uptake by APCs as well as promotion of expression of maturation markers in APCs [109, 110]. Likewise, LNP containing DOTAP and DOTMA have been shown to activate TLRs and NLRP3 inflammasome pathways [111].

Functionalization of LNP is another factor that affects their immunogenicity. For instance, T N Pham et al. demonstrated that surface functionalization of LNP with different lipid-anchored gadolinium chelates leads to complement system activation initiated by IgM antibodies [112]. Moreover, self-amplifying mRNA (SAM) vaccines encapsulated in mannosylated LNP exhibited enhanced antibody levels and Ag-specific CD8^+^ T responses compared to unglycosylated LNP [113]. Another study demonstrated that incorporating ester prodrug of anti-inflammatory steroids such as rofleponide and budesonide to LNP could decrease dose-dependent inflammatory responses induced in the case of non-functionalized mRNA-LNP formulations [114]. In addition, as shown in the case of two COVID-19 mRNA-LNP vaccines, mRNA-1273 (from Moderna) (lipid composition: SM-102, [PEG-2000]-dimyristoyl glycerol [DMG], 1,2-distearoyl-sn-glycero-3-phosphocholine [DSPC] and cholesterol), and BNT162b2 (from Pfizer/BioNTech) (lipid composition: ALC-0315: (4 hydroxybutyl) azanediyl)bis(hexane-6,1-diyl)bis(2-hexyldecanoate), ALC-0159: 2[PEG-2000]-N,N-ditetradecylacetamide, 1,2-Distearoyl-sn-glycero-3-phosphocholine (DSPC) and cholesterol), the introduction of PEG into the LNP results in the formation of anti-PEG antibodies, followed by activation of the complement system, so their use in individuals with allergic reactions is challenging. Moreover, complement system activation leads to ABC phenomenon, which results in the poor therapeutic efficacy of mRNA-LNP-based vaccines [115, 116]. Likewise, Carreño et. al. observed the same pattern of anti-PEG antibodies in irrelevant LNPs from an influenza virus mRNA vaccine and demonstrated that reactivity of such anti-PEG antibodies correlated with antibody levels against low and high MW PEG. In addition, they demonstrated that the difference in the pattern of anti-PEG antibodies in serum of participants who received mRNA-1273 and BNT162b2 was not related to the difference in the structure of PEG in the two vaccines, but due to the higher dose of PEGylated lipid in mRNA-1273 compared to BNT162b2 and also how the PEG is presented by the carrier [117]. Biodegradable and non-toxic polymers such as polysarcosin, which have similar physicochemical properties to PEG, are less immunogenic and can be used as an alternative to PEG in LNP-mediated mRNA delivery [118].

## mRNA-LNP delivery and innate immune system

The most essential function of the immune system is discrimination between self and non-self, which is coordinated by numerous and diverse cells in a unique and harmonic network. The immune system has encountered different substances and molecular patterns in various sizes, forms, and surface charges [119]. Therefore, the pharmaceutical NPs may elicit innate and adaptive immune responses and alter nanomedicines' PK and safety properties. Accordingly, keeping away from the immune response induced by NPs is the rapidly growing area in the delivery of nanomedicines into tumor cells, and for access to desirable results, the immunotoxicity tests of NPs are approved by regulatory agencies in the research and development of nanomedicines [120]. Nevertheless, the assessment of the sterility of manufactured NPs is necessary. Because bacterial endotoxins contamination may cause the formation of biomolecular corona on the surface of NPs under non-sterile conditions. The contamination of NPs by bacterial endotoxins or lipopolysaccharides (LPS) could evoke an immune response when intravenously administrated via activation of TLRs which express on the monocytes, MQs, DCs, and B lymphocytes [121]. Although excipients (such as Cremophor EL, polysorbate 80) and linkers (such as PEG) which are used in the synthesis of some NPs, are immunostimulants [122].

The initial barrier of the immune system is the innate cells which rapidly recognize distinct molecular patterns such as microbial agents, molecular apoptotic alarmins, and misfolded biomolecules [123]. Recognition of molecular patterns results in the initiation of signaling cascades that could subsequently activate the adaptive immune system via soluble factors or cell-to-cell contact [124]. The innate system consists of different cellular and soluble elements such as polymorphonuclear leukocytes (as Neut), monocytes, resident leukocytes (as MQ), professional APCs (as DC), natural killer cells (NK), eosinophils, basophils, mast cells, and distinct plasma proteins (complement system) [125].

### mRNA-LNP delivery and neutrophils

Neuts, as predominant white blood cells, are polymorphonuclear cells that have an important role in acute inflammation and are the earliest immune cells recruited into tissue during acute inflammation [126]. Hwang et al. [127] demonstrated that cationic solid lipid NPs stimulated lactate dehydrogenase (LDH) release from Neuts, a biomarker for cytotoxicity. On the other hand, neutral solid lipid NPs were safe with no adverse effect on releasing LDH. They also showed that cationic solid LNP, unlike neutral solid LNP, could degranulate Neuts by phosphorylation of Janus N kinase (JNK) and led to elastase and superoxide release anion. Eventually, unlike neutral solid LNP, cationic solid LNP could stimulate NETosis, a biomarker of activated Neuts, and increase inflammation. The activation of Neuts by cationic solid LNP may be related to the cationic surfactant that is incorporated into solid LNP. In another study, Hwang et al*.* [128] investigated the interplay between cationic liposomes and human Neuts. They used two different cationic surfactants, cetyltrimethylammonium bromide (CTAB) and soyaethyl morpholinium ethosulfate (SME), for the synthesis of cationic liposomes. Firstly, they showed that CTAB-LNP dramatically decreased the cell viability of Neuts. The cytotoxicity of SME-LNP was concentration-dependent and less than CTAB-LNP and conventional liposomes, had no significant toxic effect on Neuts. They also indicated that CTAB-LNP could increase superoxide anion production while SME-LNP and simple liposomes had no significant impact on the production of superoxide anion. Activation of the NETosis also effective in CTAB-LNP, while SME-LNP could increase NET release less than CTAB-LNP. It may be related to the more critical positive zeta potential of CTAB-LNP (+ 45.6 mV) instead of SME-LNP (+ 27.9 mV).

### mRNA-LNP delivery and macrophages

MQs are mononuclear phagocytic cells originating from fetal myeloid progenitors or are frequently derived from circulating monocytes. During tissue inflammation, monocytes are recruited and migrated into inflamed tissue under the extravasation mechanism which is controlled by inflammatory mediators such as cytokines, chemokines, and complement fragments [129]. Resident classical MQs are professional phagocytes that, similar to other innate immune cells, have PRRs such as pathogen-associated molecular pattern (PAMP) and damage-associated molecular pattern (DAMP). They are connected with microbial pathogens, apoptotic cells, or various chemicals [130]. In the concept of nano-immunology, NPs could as molecular patterns are known as nanoparticle-associated molecular patterns (NAMPs) [120]. PRRs could be categorized into two groups: membrane PRRs be composed of some TLRs, scavenger receptors, and C-type lectin receptor (CLR), and cytoplasmic PRRs consist of NOD-like receptors (NLR), RIG-1-like receptor (RLR), and endosomal TLRs [131]. Recognition of ligand by PRRs could enhance the overexpression of inflammatory mediators (such as cytokines and chemokines), antimicrobial peptides (such as defensins and cathelicidin), enzymes (such as lysozyme), endothelial adhesion molecules (such as selectins) as well as costimulatory molecules (such as CD80 and 86) [132]. Cytokines such as IL-1β, IL-6, IL-12, and TNF could trigger inflammation and evoke adaptive immune responses. Chemokines such as CCL2 and CXCL8 (also called IL-8) could recruit monocytes and Neuts, respectively [133]. The avoidance of the uptake of NPs by MQs and other phagocytes is the most critical criterion in designing nanomedicines. The route of NPs administration is determinant in NPs encountering with resident classical MQ [134]. For instance, intravenously administrated NPs must be resistant to engulfing and uptaking by blood monocytes or liver, spleen, and lung MQs. Also, the oral NPs must be resistant to MQs in lamina properia and Peyer's patches. MQs and other phagocytes also express complement receptors that bind to complement fragments (or opsonins) such as C3b and iC3b. Nevertheless, MQs express receptors for the Fc region of immunoglobulins (FcRs), and immunoglobulin G (IgG) antibodies could act as an opsonin. Opsonization facilitates the phagocytosis of particles and activates MQs and other phagocytes. Therefore, the nanopharmaceutical strategies have been designed to decrease immune-mediated recognition and activation of NPs [119, 135]. The size and surface charge of NPs are other considerable criteria in recognizing and eliminating NPs by MQs. For instance, NPs larger than 100 nm are phagocyted by liver kupffer cells and splenic red pulp MQs [136]. Also, cationic LNP, which are acceptable carriers of therapeutic nucleic acids (such as DNA, mRNA, and siRNA) are immune-cytotoxic and stimulate the production of inflammatory cytokines from phagocytes [51, 137]. Furthermore, therapeutic nucleic acids may elicit immune responses via intracellular PRRs of innate immune cells.

### mRNA-LNP delivery and dendritic cells

DCs are professional APCs that express different extracellular and intracellular receptors for PAMPs, DAMPs, and NAMPs. They are sentinel cells distributed throughout epithelial surfaces such as skin (Langerhans cells), gastrointestinal, respiratory, and urogenital tract. Ags are captured, processed, and then presented by mature DCs to naive T cells in lymph nodes. It could elicit the T cell-mediated adaptive response. Therefore, MQs and DCs as the earlier immune cells encountered with NPs that administrated through intradermal, subcutaneous, and intralymphatic routes. DCs could evoke an immune response that leads to inflammation [138].

On the other hand, NPs may be applied in cancer nano-immunotherapy via delivering different pharmaceutical biomolecules such as mRNA, siRNA, or other genes into DCs. They will discuss more in the next section. Here we direct immunotoxicity of NPs concerning DCs. The frequency of mature DCs in peripheral blood is low (around 0.2% of human blood mononuclear cells) as well as the population of DCs is heterogeneous. Accordingly, isolation of DCs from blood is complex, and ex vivo manufacturing of bone marrow-derived DCs (BMDCs) or circulating monocytes may be applied to evaluate NPs immunotoxicity [139]. Barbosa et al. [140] investigated that LNP could be a suitable carrier for delivering resveratrol into DCs without toxicity. The size of LNP was below 200 nm with a highly negative zeta potential (up to − 30 mV). They showed that LNP was internalized by naïve and stimulated DCs in a dose-dependent manner. In addition, the induction of the cell response by LNP-mRNA platforms depends on the PRR pathways and DCs engaged, so the introduction of PRR ligands or mRNAs encoding T cell-polarizing cytokines into the LNP leads to the induction of Th cell subsets by these vaccines. Also, it has been suggested that one of the important strategies to overcome the inflammation caused by the LNP content of the mRNA-LNP vaccines is targeting those lacking cationic/ionizable lipids to a subset of DCs capable of eliciting an antibody response in the absence of inflammatory agents [141].

**Fig. 1:**
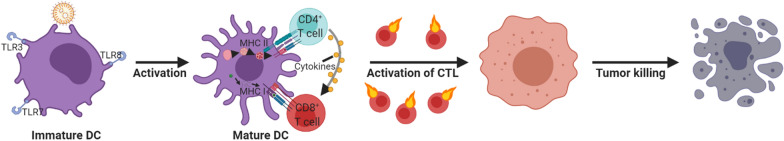
Schematic representation of the mechanism of tumor cell killing through DCs maturation by LNP assisted mRNA delivery

Furthermore, the immunotoxicity of LNP on DCs investigated by detection of apoptosis as well as morphological changes without any remarkable cytotoxicity. Finally, they showed that the immunomodulatory effects of resveratrol on DCs in the presence of inflammatory cytokine tumor necrosis factor-alpha (TNF-α) were improved by LNP. The mechanism of tumor cell killing through DCs maturation by LNP assisted mRNA delivery is represented in Fig. 1.

### mRNA-LNP delivery and complement

The complement system is one of the noticeable parts of the innate immune system that collaborates with the adaptive immune system. It comprises more than 30 proteins synthesized by the liver and released into the serum as precursor proteins or pro-enzymes (also known as zymogens) and sequentially activates each other in a cascade [142]. The main result of the complement activation is triggering inflammation, stimulating phagocytosis, and forming a membrane attack complex that leads to the elimination of pathogens. The complement system has three pathways: the classical pathway, the alternative pathway, and the lectin pathway. All pathways form a C3 convertase that cleaves C3 into C3b and C3a. C3b may bound to the microbial surface and stimulate the phagocytosis or form the membrane attack complex (C3b-9), which disregards the cell membrane and causes cell lysis. C3a (and C5a) are anaphylatoxins that recruit phagocytes such as Neuts, MQs, and eosinophils and promote inflammation [143]. Anaphylatoxins in large amounts cause a systemic allergic reaction that is mediated by Th2 cells and immunoglobulin E (IgE) antibodies. It may be referred to as complement activation-related pseudoallergy (CARPA) or non-immune allergy in the nanomedical literature [144]. Intravenously administrated NPs must be avoided from the attachment of C3b and indeed activation of the alternative pathway. For instance, there are some clinical findings of the complement activation due to the liposomal doxorubicin (Doxil^®^) [145–147]. Szebeni J listed the liposomal nanomedicines such as Amphotericin B formulations (Abelcet^®^, AmBisome^®^, and Amphotec/Amphocyl^®^), Daunorubicin (DaunoXome^®^), Doxorubicin (Doxil^®^, and Myocet^®^), and verteporfin (Visudyne^®^) which induced CARPA [144]. On the other hand, NPs in the tumor microenvironment can trigger the release of C5a and afterward cause the recruitment of regulatory and immunosuppressive immune cells such as T regulatory (T_reg_) cells which suppress the anti-tumoral immune responses [121]. Recently, Sabnis et al. [148] in Moderna Therapeutics developed a novel amino lipid series for improving PD and immunotoxicity of mRNA-based LNP. They showed for the first time that this novel amino-LNP could be tolerable at doses up to 1 mg kg^−1^ with no complement activation in both rats and primates. They also found that novel amino-LNP, compared to the conventional LNP, efficiently escaped from endosomes and released their mRNA contents into the cytosol.

## mRNA-LNP delivery and adaptive immune system

The recognition of Ags in the adaptive immune system is performed by specific T or B cell receptors. Accordingly, T cells recognize immunogenic Ags, which are presented by APCs such as DCs, MQs, and also B lymphocytes. The recognition of Ags is mediated by surface immunoglobulins of B cells and then presented by major histocompatibility complex (MHC) molecules to T cells [149]. Furthermore, the pro-inflammatory mediators such as IL-1, TNF, and IL-12 are other activatory factors of adaptive immune cells.IL-1 and IL-1ra act critical regulators of the inflammatory response to RNA vaccines. Unlike humans, murine leukocytes respond to RNA vaccines by upregulating anti-inflammatory IL-1ra relative to IL-1 (predominantly IL-1α), protecting mice from cytokinemediated toxicities at >1,000-fold higher vaccine doses. to this end, the IL-1 was unexpectedly amplified by lipid components in the formulations incorporating N1-methyl-pseudouridine-modified RNA to alleviate activation of TLR. Indeed, recognition of specific Ags by T and B lymphocytes triggers the clonal expansion of activated and memory cells. The particular traits of adaptive immune cells are specificity and memory [152]. NPs that APCs present could elicit hypersensitivities such as allergic or autoimmune responses. Attachment of LNP to proteins in blood or biological fluids and corona proteins can activate T and B lymphocytes. DCs may take up corona proteins, and these cells present the peptides to T cells. It may lead to hypersensitivity reactions [153]. In some circumstances, the chemical structure of liposomes could trigger the production of antibodies by B lymphocytes. For example, the lipid A of liposomes could produce antibodies against PC and PE in animal models. However, the titers of antibodies were not as much as the levels that cause autoimmune reactions. Moreover, repeated administration of PEGylated liposomes could induce B cells and produce anti-PEG antibodies such as IgM that accelerate their blood clearance [154].

## mRNA-LNP delivery and immunogenicity

**Fig. 2:**
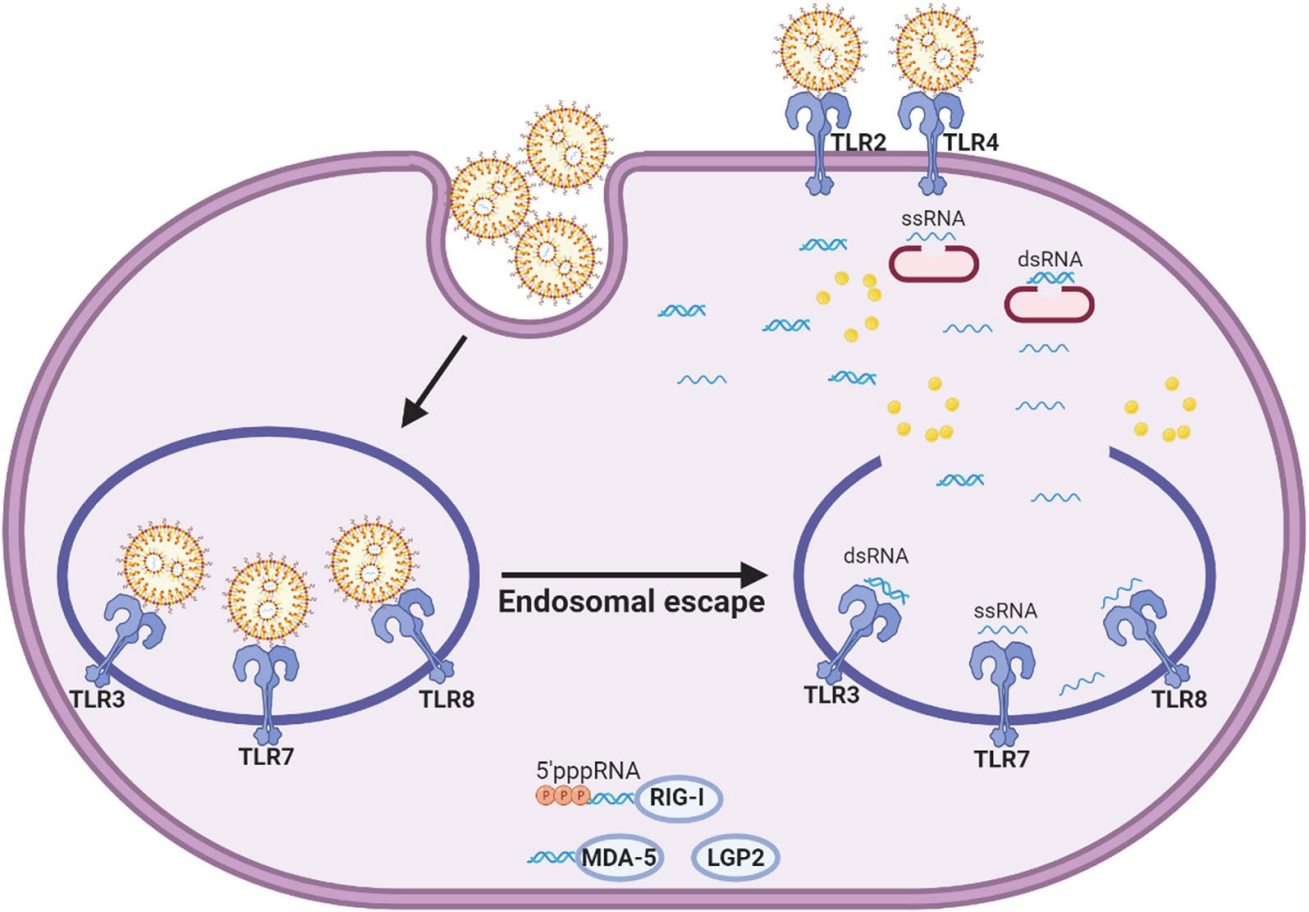
PRRs recognize both mRNA and LNP in both intracellular and extracellular pathways through TLRs, RLRs, and NLRs

The mRNA-based therapeutics open new avenues in molecular medicine, especially cancer immunotherapy. Besides biochemical instability, the immunogenicity of mRNA is the main challenge in its drug ability. Moreover, in the NPs-mediated delivery of mRNA, the escaping from endosomal degradation and releasing into the cytosol in the proper biomolecular conformation is another critical obstacle [155]. The RNA sensors of innate immune cells are endosomal PRRs and cytosolic retinoic acid-inducible RLRs. Endosomal PRRs belong to the TLRs family and include TLR 3, 7, and 8. TLR3 is a sensor for double-stranded RNA (dsRNA), and TLR 7 and 8 recognize single-stranded RNA (ssRNA). Activation of endosomal TLRs is the main initiator of overexpression of the type 1 interferons (IFN α/β), IL-1, IL-6, as well as TNF [133]. On the other hand, RLRs are retinoic acid-inducible gene I (RIG-I) and melanoma differentiation-associated gene-5 (MDA-5) that recognize viral RNAs. RIG-I recognizes a 5′-triphosphate moiety of RNA molecules, as well as MDA-5, which recognizes long double-stranded RNAs (dsRNAs) [156]. Figure 2 depicts the PRRs reorganization for mRNA and LNP in intracellular and extracellular pathways through TLRs, RLRs, and NLRs. In the extracellular pathway, TLRs as endosomal receptors are expressed by immune cells. dsRNA, which is a secondary structure form of mRNA, is detected by TLR3, and ssRNA form of mRNA is also detected by TLR7 and TLR8. The RLRs represent recognizing dsRNA through the unique interaction of uncapped dsRNA with RIG-I, binding long dsRNA with MDA-5, and binding or regulatory effects in RNA detection with LGP2. NLRP3 and NOD2 from NLRs family members detect dsRNA and ssRNA, respectively. In the intracellular pathway, cationic lipids compositions of LNP are mainly recognized by extracellular TLR2 and TLR4 and intracellular NLRP3.

Eventually, RLRs could activate the expression of IFN α/β and also, viral inhibitory proteins [157]. Therefore, the conformational modification of mRNA is one of the approaches for declining the innate immune response [158]. For instance, Kormann et al. [159] showed that partial substitution of uridine and cytidine with 2-thiouridine and 5-methyl-cytidine decreased the innate immune activation via recognition by endosomal TLRs and cytosolic RIG-1. They also found that increasing the stability of mRNA could improve the productivity of protein expression in vitro and in vivo. Besides mRNA modification, designing an accurate delivery platform is essential in avoiding an immune encounter. On the other hand, in the cancer vaccine concept, the delivery platform is designed to deliver antigen-encoded mRNA into APCs such as DCs and MQs. In this regard, using cationic LNP is applicable, but due to their possible interaction with serum opsonins, it must be considered to decline the formation of protein corona and destroyed by phagocytes. Lastly, cationic LNP may facilitate mRNA evasion from endosomes and reduce its endosomal degradation or recognition by endosomal TLRs [30]. In addition, mRNA-LNP (1273) COVID-19 vaccine, which induces a protective immune profile including induction of humoral immunity, is attended to spike-specific T follicular helper (Tfh), CD4^+^ Th1, and CD8^+^ T cells, and generation of spike-binding germinal center B cells and neutralizing antibodies [160].

In addition to mRNA, LNP used as delivery systems have been shown to cause inflammation in preclinical studies. In a study by Ndeupen et al., both intradermal and intranasal administration of LNP were shown to activate various inflammatory pathways.likewise, they produce inflammatory cytokines, resulting in a severe inflammatory response and high mortality rate in mice [161]. The cationic/ionizable lipid content of LNP is also cytotoxic and causes inflammatory responses through activating TLRs [162]. In addition, Pfizer/BioNTech and Moderna mRNA-LNP vaccines caused fever, pain, and swelling in humans [163, 164]. As a result, a balance should be struck between positive adjuvant and negative inflammatory properties of LNP in mRNA-LNP vaccines.

## Pharmacological study of mRNA-LNP delivery

The process of protein expression is managed by mRNA, which is naturally dynamic in the cytoplasm. In addition to endogenous mRNA, the exogenous synthetic mRNA can be delivered to the target cells to initiate the desired protein(s) production. The PK barriers against exogenous mRNA delivery in vitro or in vivo include degrading ubiquitous RNases in all extracellular environments and the negative charge of the cellular membrane, which repels mRNA. Both barriers reduce exogenous mRNA bioavailability and the production of the desired protein(s) [165]. Moreover, in vitro transfection of naked exogenous mRNA to humans, mice, and DCs did not represent a significant protein expression effect compared to material-mediated mRNA delivery. In the same study, the responses achieved via in vivo transfected naked exogenous mRNA were highly related to the route of administration, where the SC injection has been reported as the successful route [166]. Also, locally in vivo injection could not significantly increase the activated T-cells level [167]. Therefore, the need for improving the efficiency of in vitro and in vivo exogenous mRNA transfection has become a challenging issue. Many investigations have reported different solutions to this issue through material-free mRNA transfection methods, but the better results seem to be represented via material-mediated mRNA delivery [168]. The efficient delivery of material-mediated mRNA to DCs has been described both in vitro and in vivo subcutaneously, intravenously, and intranasally [32]. To confirm more, Pardi et al. showed significant more protein expression kinetics in mice through various intravenous (IV), intraperitoneal (IP), subcutaneous (SC), intramuscular (IM), intratumoral (IT), and intradermal (ID) routes of administration with the application of LNP for delivery of exogenous mRNA [169]. Ionizable lipid-mediated LNP not only can improve cellular uptake but also can optimize the endosomal release of the carried mRNA. In 2018, Moderna Therapeutics Company improved the pharmacological impact of mRNA-LNP with lipid manipulation in the LNP development through the synthesis of a new amino ionizable lipid, which was also utilized in their COVID-19 vaccine manufacture. Reduced endosomal trapping and sustained pharmacology and achieved optimal safety using ionizable LNP formulation in rodent and primate models [148].

### mRNA-LNP delivery in cancer types

Many investigations on cancer have recently demonstrated the benefits of mRNA-based therapies in vitro and in vivo on activating innate and adaptive immune responses, as discussed in "Impact of LNP characterization and immune responses and mRNA-LNP delivery and innate immune system" section. Here we review the impact of mRNA-based interventions on different types of cancers, including melanoma, lymphoma, hepatocellular carcinoma, colon cancer, breast cancer, and prostate cancer (Table 1).

**Table 1:** mRNA-LNP delivery in cancer types

LNP name	mRNA	Cancer type	Pharmacological effects and feedback	Refs.
Cationic LNP	OVA and the control EGFP mRNA	Lymphoma	Significant increase in expression of CD80 and CD86 in the splenic CD11^+^ T-lymphocytes Increased CD69^+^ T-cells of the spleen and the lymph nodes was the other sign of activation of T-cell responses	[174]
Ionizable lipid-based NPs (OF-Deg-Lin)	Human factor VIII (hFVIII) mRNA		Successful induction of protein expression and production in the B-cells of the spleen	[184]
LNP	IL-12A mRNA	Hepatocellular carcinoma	Enhancement of activated CD44^+^ immune cells such as CD3^+^ and CD4^+^ Th cells following IL-12 expression, shrinkage of tumor size, and increased survival in mice	[185]
cKK-E12 LNP	in vitro-transcribed mRNA was designed and formulated by cKK-E12 LNP to produce trastuzumab, an anti-HER2 monoclonal antibody	Breast cancer	In vivo PK characteristics of trastuzumab mRNA vs. synthetic trastuzumab (Herceptin^®^) in C57BL/6 mice: the significant higher serum level of trastuzumab, and interestingly the used dosage of this system was lower than Herceptin^®^, the higher morbidity-free survival in HER2^+^ mice and the lower average tumor volume via trastuzumab mRNA	[180]
PEG-coated hybrid of a cationic lipid-like compound (G0-C14) and poly lactic-co-glycolic acid (PLGA)	PTEN mRNA	Prostate cancer	Decrease in tumor size and weight with the least adverse effects on body weight increased apoptosis and declined survival of tumor cells	[36]

As cancer with high somatic mutations, melanoma has been the area of interest for mRNA-based LNP-assisted investigations [170]. The mRNA encoding the human melanoma-associated antigen recognized by T cells 1 (MART1), a specific tumor antigen, mRNA encoding Lysosome Associated Membrane Protein-1 (LAMP1), and Bax-mRNA have been used for immunization of the mice model of melanoma using different LNP. Modified liposomes such as histidylatedor mannosylated lipopolyplexes carrying mRNA vs. sugar-free lipopolyplexes lead to enhanced antitumor immune responses and increased survival rate [52, 171]. Moreover, cationic liposomes containing Bax-mRNA vs. Bax-plasmid cationic liposomes demonstrated more efficient anti-tumor effects in vitro and in vivo [172]. Oberli et al. [14] used OVA mRNA-lipid based formulation as a vaccine system to stimulate the immune system via improved CD8^+^ T-cell responses against mice models of melanoma. The usage of 3β-[N-(N′, N′-dimethylaminoethane)-carbamoyl] cholesterol hydrochloride in substitution for cholesterol did not preferably stimulate CD8^+^ T-cell toxicity. Also, the pegylated lipids with the smallest size stimulated the most clonal expansion of CD8^+^ T-cells.

On the other hand, the addition of sodium lauryl sulfate improved the performance efficacy of the LNP. The detailed characteristic properties of the LNP formulations that caused higher clonal expansion and function of the CD8^+^ T-lymphocytes included the lower molar composition of the ionizable lipid and LNP with zeta potentials of − 15 to − 3 mV. The delivery of Cre-recombinase-mRNA through the final ideal LNP to Ai14D reporter mice clarified the protein expression rate to DCs, Neuts, MQs, and B cells, respectively. Moreover, unmodified vs. modified OVA mRNA denoted a better CD8^+^ T cell production profile primarily through induction of IFN when assessed on the 7th day of the injection. In another study, OVA mRNA modified liposomes that contained α-Galactosylceramide as an immune adjuvant and agonist for invariant NKs, showed a significant reduction of tumor growth in B16-OVA melanoma model; however the survival rate was moderate [173].

Non-Hodgkin’s lymphoma is known as cancer with dysregulation of B cells, which negatively affects the activation and function of T-cells. Therefore, targeting the lymphocytes with the delivery of specific mRNAs to them could be a reasonable approach for treating this cancer. Furthermore, mRNA delivery to the spleen and lymphoid glands, as the organs of lymphocytes’ activation site, might be other targeting points [174]. Some of the studies that investigated the effects of LNP-mediated mRNA delivery in lymphoma are listed in Table 1.

An ideal anti-cancer against hepatocellular carcinoma should pass the tests for the effective anti-tumoral characteristics and have enough hepatic safety to be approved for clinical usage. LNP-mediated mRNA delivery in transgenic mice, which is the animal model of hepatocellular carcinoma with overexpressed MYC oncogene, led to shrinkage of tumor size and increased survival in these mice suggesting the possible effectiveness of mRNA with LNP. Moreover, the other important point of the mentioned delivery system was the lowest toxicity in normal cells compared to other similar works [175].

An attempt to improve mRNA delivery by LNP from PD or PK aspects is slight modifications of LNP. In some investigations on colon cancer, the complexation of protamine to liposome increased mRNA condensing to the liposome, lowered enzymatic degradation, and improved delivery efficiency. Moreover, higher immune safety and better anti-tumor activity in all IP, IT, and IV administration routes, especially the IV administration of mRNA protamine-liposome, was obtained for mRNA delivery system in comparison to DNA plasmid [176, 177].

One of the vital standard medications for treating specific breast cancers is the application of synthetic antibodies as targeted therapy. Although this treatment is an approved method, the high costs of pure antibody production, the challenging production process, low bioavailability, and high adverse reactions led the researchers to substitute the antibody synthesis with in vivo production of antibodies through the delivery of specific in vitro-transcribed mRNA [178, 179]. In vivo evaluation of PK characteristics of this system vs. synthetic trastuzumab (Herceptin^®^) in C57BL/6 mice revealed a significantly higher serum level of trastuzumab, and interestingly the used dosage of this system was lower than Herceptin^®.^ Moreover, the higher morbidity-free survival in HER2^+^ mice, the lower average tumor volume, and no significant toxic effects was observed via trastuzumab mRNA in comparison with Herceptin^®^ [180, 181].

Phosphatase and tensin homolog deleted on chromosome ten (PTEN) is a well-known tumor suppressor whose non-functionality in several cancers, such as prostate cancer, has been recognized. This event leads to dysregulation of the phosphoinositide 3-kinase (PI3K) signaling pathway, increases survival, angiogenesis, and metastasis, and declines apoptosis [182]. Therefore, PTEN mRNA delivery to prostate cancer cells could effectively increase tumor suppression potential by impacting various cell behaviors. It has been demonstrated that PEG-coated polymer-lipid hybrid NPs can be used as stable non-immunogen systems for in vitro and in vivo delivery of modified PTEN mRNA, increasing PTEN expression and early apoptosis and also inhibition of cell viability and PI3K pathway activity. Also, the PC3 tumor-bearing nude mice could achieve this delivery system intravenously with acceptable serum stability and PK properties such as significant tumor accumulation. Pharmacodynamically, this delivery system could decrease tumor size and weight with the least adverse effects on body weight, reflecting increased apoptosis and declined survival of tumor cells [36, 183].

### mRNA-LNP delivery in infectious disease

mRNA vaccines have been developed as a new technology for combating infectious diseases due to their advantages, such as ease of manufacturing, acceptable immunogenicity, and good safety profile. Moreover, mRNA vaccines are more appropriate for targeting diseases via high genetic instability and infectivity than other vaccines. mRNA-based vaccines were first used as a therapeutic drug in 1989 [186, 187]. It has been proven that the encapsulation of mRNA in LNP facilitates the control of biodistribution as well as cell targeting. Recently, six COVID-19 vaccines based on mRNA encapsulated in LNP have entered clinical trials, and mRNA-1273 (Moderna vaccine) and BNT162b2; Comirnaty (Pfizer/BioNTech vaccine) were given FDA emergency use authorization (EUA) in 2020 [188]. In a longitudinal cohort, the T cell responses were evaluated following the administration of the SARS-CoV-2 mRNA vaccine in naive and recovered individuals, and it was observed that vaccine-induced CD4^+^ and CD8^+^ T cells were similar to those of the baseline in natural infection with SARS-CoV-2. In addition, it was shown that the CD4^+^ T cell responses were rapidly induced in naive subjects following the administration of the first dose of the vaccine, and robust changes in CD4^+^ T cell response followed by the administration of the second dose, while the second dose appears to have minimal effect on the CD4^+^ and CD8^+^ T cell responses in recovered individuals [189]. In addition to SARS-CoV-2, mRNA-LNP vaccine candidates for various viruses, including HIV, seasonal influenza, Zika virus, respiratory syncytial virus (RSV), and Epstein-Barr virus (EBV), are also in the preclinical and clinical stages (Table 2) [190]. For example, several studies demonstrated that mRNA-based influenza vaccines elicit a significantly protective immune response against heterosubtypic and homologous influenza viruses [8, 191]. In addition to being utilized as a vaccine, mRNA could also be used for therapeutic goals. For instance, Pardi et al. demonstrated that the administration of mRNA encoding the anti-HIV antibody encapsulated in LNP represents passive immunotherapy against HIV-1 [192]. It should be noted that the type of mRNA delivery system as well as the administration route affect its in vivo biodistribution and, consequently its efficacy. In a study, mRNA delivery to the APCs was performed using two types of delivery systems, mRNA-LNP, and DC-mRNA, and it was observed that after IV administration, DC-mRNA has the highest accumulation in the lungs, while mRNA-LNP accumulated in the spleen, indicating its rapid passage through the lungs and further uptake by the APCs [193]. In another study, Zhang et al., evaluated the biodistribution of FLuc mRNA-LNP in mice inoculated via IM, SC, or intranasal routes. Following IM and SC injection, robust mRNA expression was observed in the upper abdomen and the injection site, while no signal was detected in route [194]. In another study, the effect of LNP content on pharmacological parameters of H10N8 mRNA formulated via the five ionizable lipids as LNP was investigated by determining lipid levels after IM administration. These ionizable lipids showed faster degradation in muscle, liver, and spleen compared to MC3. There was also a slight correlation in LNP performance between the IM and IV routes, which is related to the differences in the optimal physical or chemical properties of the two routes [195].

**Table 2:** mRNA-LNP delivery in infectious disease

LNP components	mRNA	Infection	Pharmacological effects and feedback	Refs.
Ionizable lipid: DSPC: cholesterol: PEG-lipid	mRNA encoding prM-E genes	Zika virus infection	High neutralizing antibody titers which conferred sterilizing immunity	[11]
Ionizable cationic lipid/phosphatidylcholine/cholesterol/PEG-lipid	mRNAs encoding the broadly neutralizing anti-HIV-1 antibody VRC01	HIV-1 infection	Increase in VRC01 antibody concentrations in the mice plasma 24 h after IV injection No increase in pro-inflammatory cytokine and type I IFN production	[192]
Ionizable cationic lipid (proprietary to Acuitas Therapeutics)/phosphatidylcholine/cholesterol/PEG-lipid	mRNAs encoding HIV-1 envelope, ZIKV prM-E, and influenza virus hemagglutinin (HA)	HIV, influenza, Zika viruses	Induction of high levels of Tfh cells and germinal center (GC) B cells Induction of potent antigen-specific CD4^+^ T cell responses and neutralizing antibody responses in nonhuman primates and mice	[196]
Ionizable lipid: DSPC: cholesterol: PEG-lipid	RSV F-expressing mRNAs	RSV infection	Induction of potent neutralizing antibody responses in both cotton rats and mice Induction of strong CD4^+^ and CD8^+^ T cell responses in mice	[197]
Ionizable cationic lipid (proprietary to Acuitas Therapeutics)/phosphatidylcholine/cholesterol/PEG-lipid	mRNA encoding envelope or HIV-1 Env gp160	HIV-1 infection	Induction of durable antibody responses	[198]
Ionizable cationic lipid (proprietary to Acuitas Therapeutics)/phosphatidylcholine/cholesterol/PEG-lipid	mRNA encoding the clade C transmitted/founder 1086C HIV-1 Env	HIV infection	Induction of high levels of gp120-specific antibodies in rhesus macaques and rabbits	[199]
Ionizable lipid: structural lipid: sterol: PEG-lipid	mRNA encoding the POWV prM and E genes	Powassan virus (POWV), an emerging tick-borne flavivirus	Induction of high levels of neutralizing antibodies and sterilizing immunity	[200]

Some of the clinical studies that used mRNA-LNP -based vaccines for cancer and infectious disease are summarized in Table 3.

**Table 3:** mRNA-LNP-based vaccines for cancer and infectious disease in clinical trials

Name	Condition	Clinical phase	Status	NCT No
mRNA-1345	RSV	Phase I	Recruiting	NCT04528719
Rabipur^®^	Rabies virus	Phase I	Active, not recruiting	NCT03713086
mRNA-1325 mRNA-1893	Zika virus	Phase I Phase I	Completed Completed	NCT03014089 NCT04064905
VAL-506440; mRNA-1440	Influenza A virus [H10N8]	Phase I	Completed	NCT03076385
VAL-339851; mRNA-1851	Influenza A virus [H7N9]	Phase I	Completed	NCT03345043
mRNA-1647 and mRNA-1443	CMV	Phase I	–	NCT03382405
SAM-LNP- Spike	COVID-19	Phase I	Recruiting	NCT04776317
SAM-LNP- Spike	COVID-19	Phase I	Active, not recruiting	NCT04758962
ChulaCov19 mRNA vaccine	COVID-19	Phase I	Not yet recruiting	NCT04566276
SARS-CoV-2 mRNA vaccine	COVID-19	Phase III	Not yet recruiting	NCT04847102
TAA mRNA	Melanoma	Phase I	Recruiting	NCT02410733
TAA and neo‑Ag mRNA	Breast cancer	Phase I	Recruiting	NCT02316457
mRNA-2752	Solid tumors	Phase 1	Recruiting	NCT03739931
mRNA-2416	Solid tumor, Lymphoma, and Ovarian	Phase I-II	Recruiting	NCT03323398
mRNA-4157	Bladder carcinoma and NSCLC	Phase I	Recruiting	NCT03313778
mRNA-4157	Melanoma	Phase II	Recruiting	NCT03897881

## Conclusion

As seen in the recent pandemic, LNP with dual adjuvant and carrier properties can provide mRNA vaccine candidates with the beneficial aspects of increased stability, cellular uptake, and desired therapeutic effects in solid tumors and infectious diseases. Likewise, two mRNA-LNP COVID-19 vaccine platforms from Pfizer/BioNTech and Moderna were approved and administered worldwide. Although, the possible immune responses against LNP is one of the major criteria in the PK and safety properties of nanomedicine. The chemical compositions and possible microbial contamination of LNP, as well as using of immunostimulants such as excipients and route of in vivo administration, are major determinants in immunogenicity and the PK and PD yield of mRNA delivery. Furthermore, the immunogenicity of mRNA via activation of cytoplasmic RNA sensors such as TLRs and RLRs is the main challenge in its drug ability. Therefore, the conformational modification of mRNAs is one of the approaches to the decline of immune responses. Taken together, it seems likely that further research between immunology and pharmacology areas and LNP compositions will illuminate the dark side of LNP for safe and efficient mRNA delivery. Consequently, thoroughgoing studies in immunopharmacological responses of LNP for therapeutic delivery of mRNA would cause a dramatic evolution in cancer and infectious disease treatment.

## Data Availability

Not applicable.

## Abbreviations

ABC: Accelerated blood clearance
Ags: Antigens
APCs: Antigen-presenting cells
CAR: Chimeric antigen receptor
CARPA: Complement activation-related pseudoallergy
CLR: C-type lectin receptor
CME: Clathrin-mediated endocytosis
CRISPR: Clustered regularly interspaced short palindromic repeats
CTAB: Cetyltrimethylammonium bromide
DAMP: Damage-associated molecular pattern
DC: Dendritic cell
DOPC: Dioleoylphosphatidylcholine
DOPE: 1,2-Dioleoyl-sn-Glycero-3 phosphatidylethanolamine
DMG-mPEG: Dimyristoyl-rac-glycero-3-methoxy-PEG
DOTAP: 1,2-Dioleoyl-3-trimethylammonium-propane) DPPC: Dipalmitoyl-phosphatidylcholine
DSPC: 2-Distearoyl-sn-glycero-3-phosphocholine
dsRNAs: Double-stranded RNAs
EGFP: Enhanced green fluorescent protein
ID: Intradermal
IFN: Interferon
IgG: Immunoglobulin G
IgE: Immunoglobulin E
IgM: Immunoglobulin
IM: Intramuscular
IP: Intraperitoneal
iPSCs: Induce pluripotent stem cells
IT: Intratumoral
IV: Intravenous
JNK: Janus N kinase
LDH: Lactate dehydrogenase
LNP: Lipid nanoparticles
LPS: Lipopolysaccharides
MART1: Melanoma associated antigen recognized by T cells 1
MDA-5: Melanoma differentiation-associated gene-5
MHC: Major histocompatibility complex
mPEG-DSPE: Methoxy-PEG-distearoyl phosphoethanolamine
MPS: Mononuclear phagocyte system
mRNA: Messenger RNA
MQ: Macrophage
MZ: Marginal zone
NAMP: Nanoparticle-associated molecular pattern
MBL: Mannose-binding lectin
NET: Neutrophil extracellular traps
Neut: Neutrophil
NK: Natural killer cell
NLR: NOD-like receptor
NPs: Nanoparticles
OVA: Ovalbumin
PAMP: Pathogen-associated molecular pattern
PD: Pharmacodynamics
PE: Phosphatidylethanolamine
PEG: Polyethylene glycol
PI3K: Phosphoinositide 3-kinase
PK: Pharmacokinetics
PLGA: Poly lactic-co-glycolic acid
PRR: Pattern recognition receptor
PTEN: Phosphatase and tensin homologue deleted on chromosome 10
RNAi: RNA interference
RLR: RIG-1-like receptor
RIG-I: Retinoic acid-inducible gene I
siRNA: Short interfering RNA
ssRNA: Single-stranded RNA
SME: Soyaethyl morpholinium ethosulfate
SC: Subcutaneous
TALEN: Transcription activator-like effector nuclease
TLR: Toll-like receptor
TNF-α: Tumor necrosis factor alpha
Th2: T-helper 2
T_reg_: T regulatory
ZFNs: Zinc-finger nucleases
